# Advanced biomaterials in pressure ulcer prevention and care: from basic research to clinical practice

**DOI:** 10.3389/fbioe.2025.1535588

**Published:** 2025-02-17

**Authors:** Shaoqiang Tian, Wei Bian

**Affiliations:** ^1^ Department of Emergency Medicine, The First People’s Hospital of Shenyang, Shenyang, China; ^2^ Department of Neurosurgery, The First People’s Hospital of Shenyang, Shenyang, China

**Keywords:** pressure ulcers, hydrogels, alginate, chitosan, PLGA, polyurethane

## Abstract

Pressure ulcers are a common and serious medical condition. Conventional treatment methods often fall short in addressing the complexities of prevention and care. This paper provides a comprehensive review of recent advancements in advanced biomaterials for pressure ulcer management, emphasizing their potential to overcome these limitations. The study highlights the roles of biomaterials in enhancing wound healing, preventing infections, and accelerating recovery. Specific focus is placed on the innovation and application of multi-functional composite materials, intelligent systems, and personalized solutions. Future research should prioritize interdisciplinary collaboration to facilitate the clinical translation of these materials, providing more effective and tailored treatment approaches. These advancements aim to improve the quality of life and health outcomes for patients by offering more reliable, efficient, and patient-specific therapeutic options.

## Highlights

• This review summarizes recent progress in advanced biomaterials for pressure ulcer prevention and treatment, from research to clinical use.
• Multi-functional and intelligent biomaterials hold great promise for improving wound healing and infection prevention.
• Future research highlights the need for interdisciplinary collaboration and personalized strategies to advance clinical applications.

## 1 Introduction

Pressure ulcers (PUs), also known as pressure sores or bedsores, are areas of tissue damage caused by prolonged pressure on bony parts of the body like the sacrum, hips, heels, and elbows (Mervis and Phillips, 2019). This pressure restricts blood flow, leading to tissue oxygen loss, ischemia, and eventually, necrosis. While constant pressure is the main cause, other factors like shear forces and friction also play significant roles in PU development (Blackburn et al., 2020). PUs are a major healthcare issue, especially among the elderly (Wei et al., 2021), people with limited mobility (Sprigle et al., 2020), and those confined to bed for long periods (Deng et al., 2024). In China, a meta-analysis found the incidence of PUs among the elderly to be 12.77% (Hu et al., 2020). This highlights the widespread nature of the problem and the differences in prevention and treatment practices across care settings. Globally, the incidence of PUs is increasing due to aging populations and the growing prevalence of chronic illnesses. This trend significantly impacts both patients' quality of life and healthcare systems. PUs cause intense pain and discomfort, severely affecting patients' daily lives and mental health (Qian et al., 2024). The long healing process often leads to frustration, helplessness, and at times, depression. Infections are another serious risk (Malone and Schultz, 2022), which can threaten patients’ lives (Hajhosseini et al., 2020). For bedridden individuals, PUs further reduce mobility and independence, worsening their overall quality of life. The economic impact of PUs on healthcare is considerable. Treatment often requires specialized care, advanced wound dressings, and equipment like negative pressure wound therapy. Severe cases involving infection or significant tissue damage may need surgery, increasing costs even more. In the United States, annual PU-related expenses run into billions of dollars. PUs also lengthen hospital stays and raise readmission rates, adding further strain to healthcare systems.

Conventional treatments for pressure ulcers (PUs) include pressure relief (e.g., repositioning and specialized support surfaces), wound dressings, and pharmacological interventions (BoykoTatiana et al., 2018). While these methods can reduce pain and help prevent infection, they often involve long treatment durations, limited effectiveness, and low cure rates. Pressure relief is essential but difficult to fully implement for patients with prolonged immobility, which limits its overall success. Traditional moist dressings, though helpful, carry a risk of infection and may not effectively promote tissue regeneration. Pharmacological treatments can manage symptoms and relieve pain, but their impact on wound healing is often slow, with the added concern of potential drug resistance. These limitations highlight the challenges of current PU management strategies in clinical practice, making it difficult to achieve complete wound resolution. The need for more advanced and effective materials and methods for PU prevention and treatment is clear. Recent research points to the potential of biomedical materials as a promising solution for improving PU outcomes. Innovations in biomaterials have introduced new approaches for managing these challenging wounds. For example, biomaterials can accelerate wound healing, prevent infections, and enhance both the speed and quality of tissue regeneration (Deng et al., 2022; Nguyen et al., 2023). Using biocompatible and pro-regenerative biomaterials is emerging as a highly effective strategy for improving PU treatment outcomes. Studies show that incorporating biomaterials into PU treatment protocols significantly enhances efficacy compared to conventional methods. These materials not only promote faster short-term healing but also provide long-term benefits, reducing treatment costs and improving patients' quality of life (Pan et al., 2023).

This review will explore the role and potential of biomaterials in PU treatment. We will examine the efficacy and advantages of various biomaterials based on recent research and discuss future directions for their development and application in PU prevention and treatment. Our goal is to provide clinicians with evidence-based, effective treatment options and offer valuable insights for future research and clinical practice in this critical area of wound care.

## 2 Classification of advanced biomaterials for pressure ulcer prevention and care

### 2.1 Hydrogels

Hydrogels are a class of biomaterials widely used in biomedicine due to their high water content, softness, and breathability (Cascone and Lamberti, 2020). These unique properties make hydrogels particularly effective for applications such as pressure ulcer (PU) management, wound healing, and drug delivery (Li et al., 2022).

The most defining feature of hydrogels is their high water content, which results from their three-dimensional network structure. This structure allows hydrogels to absorb and retain large amounts of water, creating a moist environment that is essential for cell growth and tissue regeneration during wound healing. Their hydrophilic nature helps accelerate the repair process by maintaining optimal conditions for cell migration and proliferation. The softness and elasticity of hydrogels further enhance their benefits, allowing them to conform closely to the wound bed. This adaptability ensures personalized care for wounds of different shapes and sizes while providing a more comfortable healing experience. Their soft texture also minimizes irritation and friction on healthy surrounding tissues, reducing pain and improving patient comfort. Another key advantage of hydrogels is their excellent breathability, which allows air and water vapor to pass through. This feature prevents bacterial growth, ensures proper oxygen supply, and manages moisture levels, helping to avoid complications caused by excess fluid or temperature fluctuations. Improved breathability not only supports faster wound healing but also increases comfort and wearability for patients. In addition to these physical and functional properties, hydrogels exhibit strong biocompatibility, meaning they rarely cause allergic reactions or skin irritation (Cascone and Lamberti, 2020). This makes them a safe choice for various clinical applications, including wound healing, burn care, and skincare. Their compatibility with biological tissues further underscores their effectiveness in promoting tissue repair and regeneration. By combining these advantageous properties, hydrogels represent a significant advancement in PU management and other biomedical fields, offering a versatile and patient-friendly solution.

**FIGURE 1:**
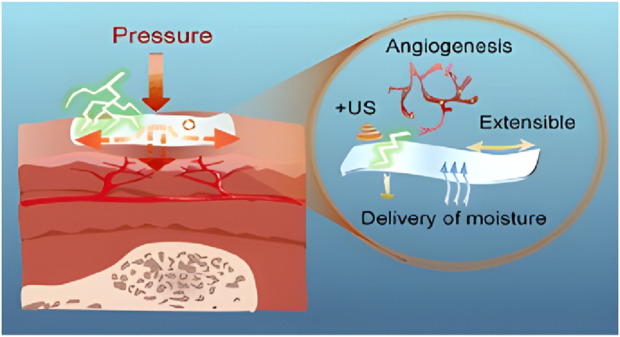
Hydrogel for Prophylaxis and Early Treatment of Pressure Injuries/Pressure Ulcers. Reproduced with permission from Li et al. (2022). Copyright ^©^ 2022 The Authors.

**FIGURE 2:**
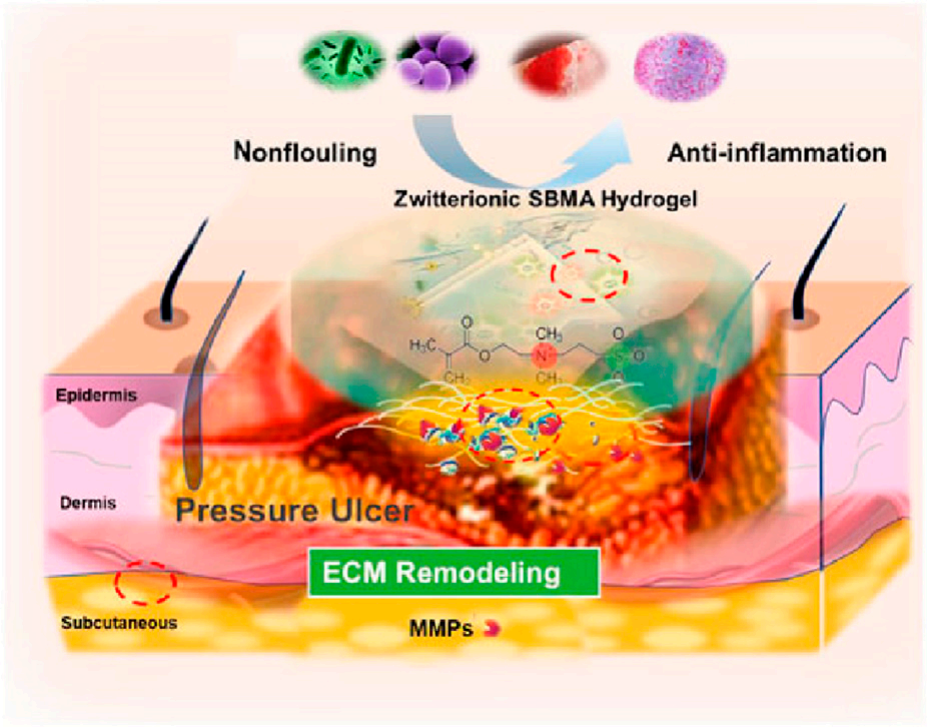
Hydrogel Activates Autophagy to Promote Extracellular Matrix Remodeling for Improved Pressure Ulcer Healing. Reproduced with permission from Li et al. (2021). Copyright ^©^ 2021 The Authors.

The use of hydrogels as pressure ulcer (PU) dressings offers several key benefits, including wound hydration, pressure relief, and enhanced healing (Figures 1, 2). Studies have shown that hydrogel dressings significantly improve PU management by maintaining a moist wound environment, which prevents desiccation and supports cell growth and tissue regeneration. This moist environment not only reduces healing time but also creates optimal conditions for wound repair. Hydrogel dressings also help alleviate pressure on the affected area. Their softness and elasticity allow them to conform to the wound’s contours, effectively distributing pressure and minimizing further tissue damage. By reducing friction and shear forces, they help prevent additional breakdown of surrounding tissues. In addition, the biocompatibility of hydrogels ensures close adherence to the wound surface, creating an ideal environment for healing. Their hydrating properties stimulate tissue regeneration and repair, accelerating the healing process and improving overall treatment outcomes. These combined advantages make hydrogel dressings a valuable tool in PU management.

In conclusion, hydrogel dressings are a highly effective and versatile option for managing pressure ulcers. Their ability to maintain a moist wound environment, relieve pressure, and support tissue regeneration has made them a valuable tool in clinical practice. By creating optimal conditions for healing, hydrogel dressings can accelerate recovery, improve treatment outcomes, and enhance the quality of life for patients with pressure ulcers.

### 2.2 Nanomaterials

Nanomaterials are increasingly being used in medicine, with nano-silver and nano-zinc oxide standing out for their strong antibacterial properties (Du, 2024). These materials exhibit broad-spectrum activity against bacteria, viruses, and fungi, making them valuable components in medical devices, wound dressings, antimicrobial coatings, and similar products.

Nano-silver’s high surface area-to-volume ratio enhances its reactivity, allowing it to release silver ions that disrupt bacterial cell walls and membranes, ultimately causing bacterial death (Du, 2024). This mechanism provides effective antimicrobial activity, including against drug-resistant strains. Nano-silver also targets viral outer membranes and genetic material, preventing replication and spread. These properties make it essential for infection control in medical devices, personal protective equipment, and other healthcare tools. Additionally, nano-silver’s antifungal activity helps combat mold and fungal infections, broadening its utility in clinical and environmental applications. Nano-zinc oxide, known for its photocatalytic properties (Chopra, 2022), produces reactive oxygen species when exposed to ultraviolet (UV) light. These reactive molecules effectively eliminate bacteria and viruses, making nano-zinc oxide valuable for applications such as environmental remediation and water purification. In sunscreen products, it provides physical UV protection while also delivering antibacterial benefits, reducing the risk of skin infections caused by sun exposure. Furthermore, nano-zinc oxide’s anti-inflammatory properties help soothe skin irritation and promote wound healing, making it an ideal component in wound dressings and topical ointments.

**FIGURE 3:**
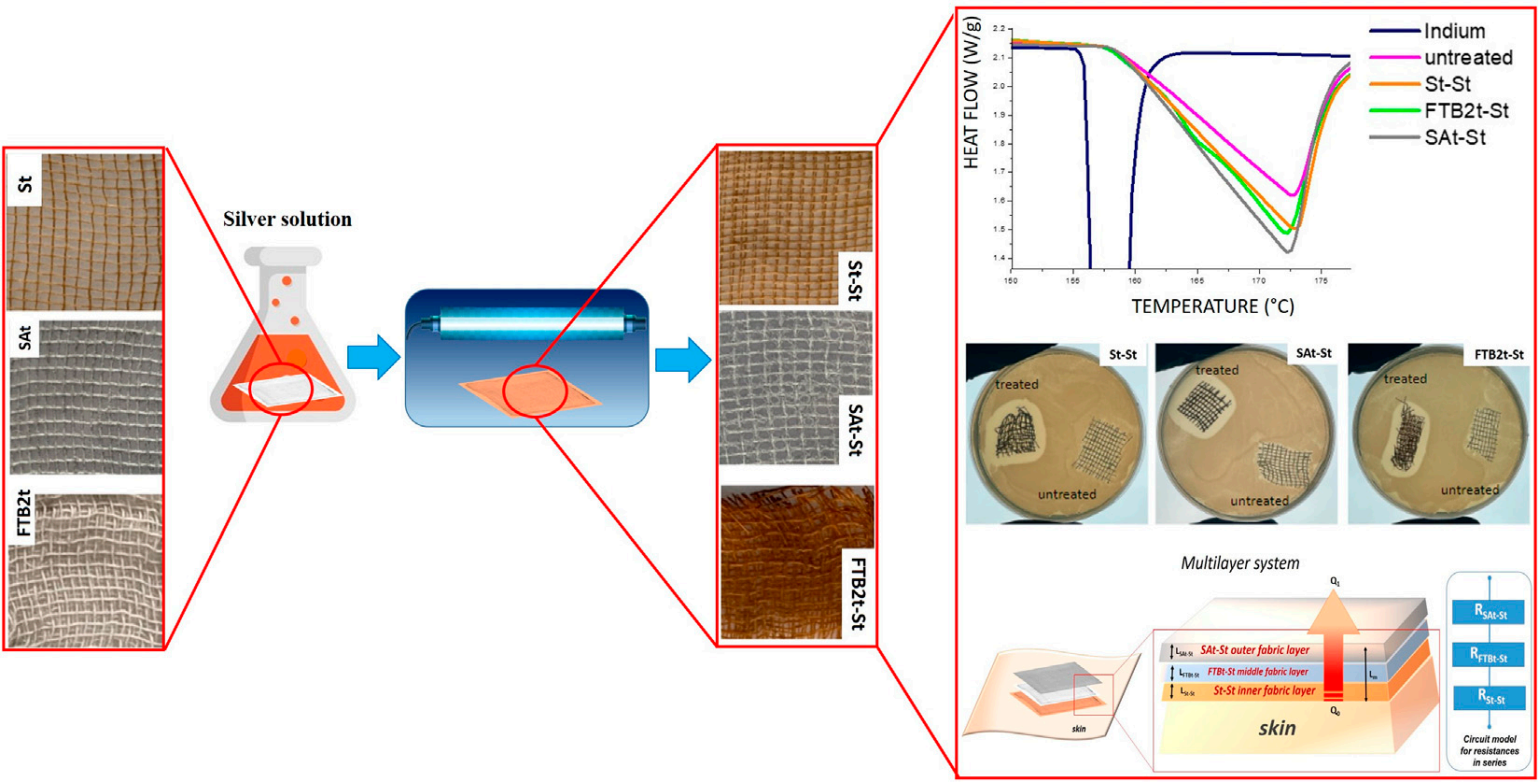
Nanotechnological Antibacterial Wound Dressings for Pressure Ulcer Prevention. Reproduced with permission from Pollini et al. (2024). Copyright ^©^ 2024 The Authors.

Studies have shown that pressure ulcer (PU) dressings containing nano-silver or nano-zinc oxide can accelerate wound healing, reduce infection risk (Figure 3), and improve overall treatment outcomes (Pollini et al., 2024; Rybka et al., 2022). The antibacterial and anti-inflammatory properties of these nanomaterials play a key role in promoting tissue repair and regeneration. Additionally, dressings incorporating nanomaterials offer favorable features such as conformability, absorbency, and breathability, which help maintain optimal wound moisture and create an ideal healing environment. These dressings are also easy to apply and comfortable for patients, further enhancing their practicality in clinical use.

While the benefits of nanomaterials in PU dressings are well-documented, ongoing research is focused on evaluating potential side effects and long-term safety. Current evidence indicates that, when used at appropriate concentrations, these nanomaterials do not pose significant risks. Overall, the integration of nanomaterials into PU dressings provides substantial advantages, including faster healing, reduced infection rates, and improved treatment outcomes. As rigorous studies continue to validate their efficacy and safety, nanomaterial-based PU dressings are becoming increasingly accepted in clinical practice, offering more effective treatment options for patients.

### 2.3 Extracellular matrix (ECM)

The extracellular matrix (ECM) is a complex and dynamic network that surrounds cells, consisting of structural proteins, polysaccharides, and bioactive molecules (Karamanos et al., 2021). Its key components include collagen, fibronectin, and elastin, which provide structural and mechanical support; polysaccharides such as sulfated glycosaminoglycans and hyaluronic acid, which contribute to hydration and matrix organization; and bioactive molecules like growth factors and cell adhesion molecules, which regulate cell behavior and tissue function. Together, these elements create a supportive framework and transmit essential biochemical and mechanical signals critical for maintaining tissue integrity and cellular activities.

The extracellular matrix (ECM) performs a wide array of functions essential for tissue health and stability. It provides structural support to tissues, facilitates cell adhesion and migration, regulates cell signaling pathways, influences cell morphology and polarization, and maintains tissue homeostasis. By mediating interactions between cells and their microenvironment, the ECM supports cell survival and function, playing a critical role in tissue repair, regeneration, and stability. Understanding ECM composition and function is fundamental for advancing tissue engineering, disease treatment, and related fields. In tissue engineering, the ECM is particularly important for the development of skin substitutes, offering innovative solutions for pressure ulcer (PU) prevention and treatment (Sheikholeslam et al., 2018). ECM-based biomaterials mimic the structure and biological functions of native skin, creating an ideal environment for wound repair. Studies demonstrate that ECM-based skin substitutes can significantly accelerate PU healing by serving as a structural scaffold for cells and delivering a rich array of growth factors and signaling molecules. These elements stimulate cell migration and proliferation, promoting tissue reconstruction (Diller and Tabor, 2022). Additionally, the ECM enhances the wound microenvironment by maintaining a moist environment that prevents desiccation and infection. Antimicrobial components such as sulfated glycosaminoglycans inhibit bacterial growth, while the ECM’s ability to regulate wound pH creates conditions conducive to healing. Moreover, angiogenic factors like vascular endothelial growth factor (VEGF) and fibroblast growth factor (FGF) promote angiogenesis, improving blood supply to the wound and ensuring the delivery of nutrients and oxygen critical for tissue repair. ECM-based skin substitutes also play a preventative role in high-risk populations. By acting as a protective barrier, they reduce friction and shear forces over vulnerable areas, effectively lowering the risk of PU development. The combination of these properties makes ECM-based biomaterials highly versatile and effective in wound care.

In summary, ECM-based skin substitutes show immense potential for transforming PU prevention and treatment. Their ability to replicate native skin functions and enhance wound healing offers promising opportunities for clinical practice. Continued research and optimization are crucial to fully harness their benefits and further improve outcomes in PU management.

### 2.4 Smart biomaterials

Recent advances in smart materials, particularly temperature-sensitive and pH-sensitive types, have unlocked new possibilities for pressure ulcer (PU) monitoring and treatment (Tang et al., 2024; Mu et al., 2024). Temperature-sensitive materials can reversibly alter their physical and chemical properties, such as volume, shape, and wettability, in response to temperature fluctuations. This behavior is driven by their phase transition near a critical temperature, known as the lower critical solution temperature (LCST), which is triggered by the interaction of their hydrophilic and hydrophobic groups (Kotsuchibashi, 2020; Khutoryanskiy and Georgiou, 2018). Similarly, pH-sensitive materials respond to changes in proton concentration, modulating properties like solubility and ionization state. These characteristics make pH-sensitive materials highly adaptable to varying microenvironments, particularly in wounds where pH levels can fluctuate significantly (Ofridam et al., 2021). The intelligent and dynamic behavior of both temperature-sensitive and pH-sensitive materials makes them highly promising for biomedical applications, including advanced wound care and PU management.

Research has shown that skin temperature at pressure ulcer (PU) sites is significantly elevated, making temperature-sensitive materials valuable tools for monitoring PU development and progression in real time. Flexible sensor pads integrating multiple sensors can continuously and non-invasively track PU-related vital signs, providing critical data for early prevention (Kaewjamnong and Hongwitayakorn, 2021). For instance, Gillard et al. explored the relationship between temperature and blood flow in PU detection, demonstrating how temperature sensors can improve early diagnosis by continuously monitoring changes in pressure and temperature (Gillard et al., 2023). Similarly, pH-sensitive materials offer significant potential for assessing PU status. PU wounds often exhibit pH variations, and pH-sensitive materials can act as colorimetric indicators, providing visual feedback on wound conditions. Advanced pH-sensing fabric sensors, based on flexible wearable technology, can continuously monitor wound pH fluctuations, offering clinicians valuable real-time data for wound assessment (Du and Ciou, 2019). Additionally, pH-sensitive hydrogels can respond to these changes by modulating the release of antimicrobials or growth factors, creating a favorable wound environment and accelerating PU healing (Haidari et al., 2021).

The evidence highlights the transformative potential of smart materials, such as temperature- and pH-sensitive technologies, in advancing PU monitoring and treatment. These materials enable real-time tracking of PU development, support early prevention strategies, and enhance clinical assessment. Furthermore, by tailoring their properties, they can actively promote wound healing and improve patient outcomes. Future research aimed at optimizing the performance of these materials, alongside advancements in flexible electronics and wearable sensing technologies, holds great promise for addressing the complex challenges of PU management.

## 3 Innovative applications of biomaterials in pressure ulcer prevention and care

PUs are a common issue in patients with prolonged immobility. To address this, researchers and medical device manufacturers have developed smart mattresses and seat cushions that utilize advanced biomaterials and sensor technologies. These intelligent products monitor pressure distribution and adjust cushioning properties in real time to achieve balanced pressure dispersion, providing relief and effectively preventing PUs. Smart mattresses incorporate pressure-sensitive sensors, adjustable hardness materials, and breathable materials to optimize patient care. Pressure-sensitive sensors accurately measure tissue pressure and transmit data to a control system for analysis (Silva et al., 2021; Yang et al., 2021). This data enables the dynamic adjustment of mattress firmness using adjustable hardness materials, which provide targeted support and pressure relief where needed (Kumagai et al., 2019). Additionally, breathable materials enhance the skin microenvironment by promoting air circulation and reducing moisture buildup, key factors in PU prevention (Das and Baker, 2016). By continuously monitoring pressure fluctuations and responding in real time, smart mattresses and seat cushions not only prevent the formation of PUs but also mitigate their progression. The integration of biomaterials and sensor technologies in these devices represents a significant advancement in PU management, offering innovative solutions for improving patient outcomes (Memari et al., 2021; Yang et al., 2024; Ghosh et al., 2024).

Similarly, smart seat cushions are designed for patients who sit for extended periods, focusing on reducing pressure on the sacral area to prevent nerve damage and pressure ulcers (Hepburn et al., 2017). Key biomaterials used in these cushions include shape memory foam, pressure-relief contour designs, and position-sensing sensors. Shape memory foam conforms to the patient’s body contours, evenly distributing pressure and providing optimal support (Kumar et al., 2019). Contour designs help minimize sacral pressure and reduce skin friction, while position-sensing sensors continuously monitor sitting posture and pressure distribution. These sensors enable real-time structural adjustments, enhancing support and adaptability. Together, these features make smart seat cushions highly effective in preventing sacral pressure ulcers and improving patient comfort and quality of life. In conclusion, the integration of biomaterials into smart mattresses and seat cushions represents an innovative approach to pressure ulcer prevention and treatment. These intelligent devices provide real-time monitoring and pressure redistribution, significantly reducing the risk of pressure injuries caused by prolonged immobility. As biomaterial technology continues to advance, smart mattresses and cushions are becoming indispensable clinical tools. Additionally, specialized garments and wearable devices incorporating biomaterials further enhance PU prevention by providing targeted pressure relief and skin protection for patients confined to prolonged bed rest or sitting (Salgueiro-Oliveira et al., 2023; Barboza et al., 2022).

The integration of sensor technology with biomaterials marks a significant advancement in the development of intelligent medical devices for pressure ulcer (PU) prevention and treatment. These devices continuously monitor pressure distribution, movement, and skin health to assess and mitigate the risks of PU formation. Sensors track pressure levels, posture changes, and activity, while also collecting data on skin health indicators such as temperature, humidity, and pressure. This comprehensive monitoring enables dynamic adjustments in material hardness and shape based on real-time feedback, ensuring optimal pressure relief and support. By tracking patient movement, these devices can quickly detect periods of prolonged immobility and prompt necessary positional adjustments. Skin health monitoring provides immediate feedback on critical factors like temperature and humidity, enabling early interventions to prevent tissue damage. Additionally, continuous data recording and analysis support the creation of personalized PU prevention plans tailored to individual patient needs. This innovative approach allows for more precise and proactive care, significantly reducing the risk of pressure ulcers and enhancing patient quality of life. As sensor and biomaterial technologies continue to advance, these intelligent medical devices have the potential to transform healthcare by improving outcomes for patients at risk of PUs. Such innovations represent a crucial step toward more effective, personalized, and technology-driven medical care.

## 4 Advanced biomaterials as wound dressings for pressure ulcer prevention and care

The preceding section provided a general classification of advanced biomaterials for pressure ulcer prevention, highlighting categories such as hydrogels, nanomaterials, ECMs, and smart biomaterials. Building on this framework, this section will explore specific biomaterials, focusing on their applications in wound dressings. Key examples include alginate and chitosan, which demonstrate significant potential in PU prevention and care. This transition from a broad overview to specific case studies aims to illustrate the practical implementation of these biomaterials in enhancing wound management strategies.

### 4.1 Alginate

Alginate wound dressings have proven to be highly effective and widely used in the clinical management of pressure ulcers (Hill et al., 2022; Dong et al., 2024; Ausili et al., 2013). As a biodegradable material, alginate offers excellent biocompatibility, minimizing secondary damage and pain for patients with chronic wounds (Hurtado et al., 2022; Sahoo and Biswal, 2021). Its superior absorptive capacity efficiently manages wound exudate, creating a dry and clean environment that reduces infection risk and supports healing (Zhang and Zhao, 2020; Barros et al., 2021). These qualities make alginate dressings particularly well-suited for pressure ulcer treatment. In addition to their absorptive properties, alginate dressings exhibit antimicrobial and anti-inflammatory effects, combating infection, reducing inflammation, and promoting faster wound healing (Varaprasad et al., 2020; Huang et al., 2022). Their conformable nature allows them to be tailored to the wound’s specific shape and size, ensuring optimal coverage and protection while preventing bacterial ingress (Op’t Veld et al., 2020). The ease of application and removal simplifies dressing changes, reducing discomfort for patients and lightening the workload for healthcare providers. Clinically, alginate wound dressings not only alleviate pressure on vulnerable areas but also promote healing, making them an indispensable tool for both the prevention and treatment of pressure ulcers. Their combination of effectiveness, patient comfort, and convenience has made them a preferred choice among healthcare providers and patients alike.

Several commercially available products utilize alginate for effective pressure ulcer management. Purilon^®^ (Coloplast) is a gel containing sodium carboxymethyl cellulose and calcium alginate, specifically designed for necrotic and sloughy wounds. It promotes debridement and hydration, creating an optimal wound environment for healing (Wang et al., 2023; Dong et al., 2020). Askina Calgitrol Ag (B Braun Hospicare Ltd.) is an advanced alginate silver dressing with a bi-layer design. It combines an exudate-absorbing polyurethane foam layer with a contact layer made of an alginate matrix embedded with silver ions. In a moist environment, the silver ions are gradually released, leveraging their antimicrobial properties. This dressing also benefits from the high absorptive capacity of calcium alginate and polyurethane foam, making it suitable for treating pressure ulcers ranging from grade I to IV, with demonstrated clinical efficacy (Ricci et al., 2008; Addison et al., 2005). Another example, Urgosorb™, combines calcium alginate fibers with hydrocolloid technology to address sloughy and granulating wounds with moderate to high exudate levels. This unique composition helps effectively manage exudate while supporting wound healing (Stevens and Chaloner, 2005).

The clinical effectiveness of alginate dressings in pressure ulcer management has been well-documented. Belmin et al. (2002) conducted a study comparing a sequential strategy using calcium alginate followed by hydrocolloid dressings to hydrocolloid dressings alone in treating grade III or IV pressure ulcers. In this study, 110 elderly patients were randomized to either the sequential approach (calcium alginate dressing [UrgoSorb] for 4 weeks followed by a hydrocolloid dressing [Algoplaque] for another 4 weeks) or the control group (hydrocolloid dressing [Duoderm E] for the full 8 weeks). Pressure ulcer surface area was measured weekly, with primary endpoints including the mean absolute surface area reduction (SAR) over 8 weeks and the proportion of patients achieving at least a 40% reduction (SAR40). Patients in the sequential group showed significantly better outcomes, with 68.4% versus 22.6% (p < 0.0001) achieving SAR40 at 4 weeks, and 75.4% versus 58.5% (p < 0.0001) by 8 weeks, compared to the control group. These results suggest that the sequential use of calcium alginate and hydrocolloid dressings promotes faster healing in grade III or IV pressure ulcers compared to hydrocolloid monotherapy. A similar study on spina bifida patients evaluated the sequential use of calcium alginate and foam dressings. Significant improvements in wound healing were observed, with the mean ulcer surface area reducing from 12.5 ± 7.5 cm^2^ at baseline to 3.7 ± 5.2 cm^2^ at 12 weeks (p < 0.001). Additionally, 75% of patients achieved a 50% reduction in surface area by the end of the study (Ausili et al., 2013). Together, these findings highlight the potential of sequential strategies incorporating calcium alginate dressings to enhance pressure ulcer treatment outcomes across different patient groups.

However, a review by Dumville et al. (2015) concluded that current evidence does not definitively support the superiority of alginate dressings over other dressings, topical treatments, or interventions for healing pressure ulcers. Importantly, the trials included in this review were often small, short-term, and susceptible to bias, resulting in low or very low quality evidence. Therefore, larger, more robust studies with longer follow-up periods are needed to definitively establish the efficacy of alginate dressings in pressure ulcer management.

### 4.2 Collagen

Collagen-based wound dressings have shown great potential and are widely used in managing pressure ulcers (Graumlich et al., 2003). These dressings offer a comprehensive approach to wound care by combining biocompatibility, healing promotion, antimicrobial activity, and flexibility.

Collagen’s natural biocompatibility (Liao et al., 2023a) makes these dressings suitable for a wide range of patients, especially those who are bedridden and require long-term care. As a biological material that closely resembles human tissue components (Nimni and Harkness, 2018), collagen supports cell growth and regeneration, which are essential for effective wound healing. Collagen dressings promote healing at all stages (Sharma et al., 2022), from blood clotting and cell migration to tissue regeneration. By maintaining a moist wound environment, they encourage skin cell growth, accelerate healing, and reduce recovery time. This also helps minimize the risk of complications. The antimicrobial properties of collagen dressings (Liao et al., 2023b; Neacsu et al., 2021) play a key role in keeping the wound bed clean and reducing the risk of infection. This is particularly important for patients who are immunocompromised or at high risk of infection. Finally, collagen dressings are highly conformable (Zhao et al., 2020), meaning they can easily adapt to the size and shape of the wound. This ensures optimal coverage and protection, further reducing the risk of infection and promoting efficient healing. Their customized fit also helps prevent the development of new pressure ulcers and minimizes surface infections that could delay recovery.

Collagen dressings are widely available for treating pressure ulcers, as shown in Table 1. Clinical studies suggest that these dressings accelerate wound healing, reduce infection risks, and improve the quality of life for patients with pressure ulcers.

**TABLE 1:** Commercially available collagen dressings for the treatment of pressure ulcers.

Brand	Company	Composition
Catrix^®^	Lescarden Inc	Fine white powder (bovine cartilage)
Collieva™	CollMed Laboratories	Bovine collagen (Type I)
DermaCol™	DermaRite Industries, LLC	Type I bovine collagen powder/sheet
Fibracol^®^	Systagenix	90% collagen and 10% alginate
Promogran™	Systagenix	55% collagen (bovine), 45% oxidized regenerated cellulose (ORC)
Promogran Prisma^®^	Systagenix	44% oxidized regenerated cellulose, 55% collagen and 1% silver-ORC
Primatrix^®^	Integra life sciences	Fetal bovine dermis
Biostep™ Ag	Smith and Nephew, Inc	A silver collagen matrix dressing with calcium alginate and ethylenediaminetetraacetic acid

In a randomized controlled trial, Graumlich et al. compared collagen injections (Type I collagen, Medifil, Kollagen, BioCore, Topeka, KS) with hydrocolloid therapy (DuoDerm, ConvaTec, Princeton, NJ) in 65 patients with stage II or III pressure ulcers (Graumlich et al., 2003). Thirty-five patients received daily collagen injections, while 30 received hydrocolloid dressings twice weekly. The primary outcome was the rate of complete healing within 8 weeks, while secondary outcomes included time to healing and the daily healed ulcer area. Results showed similar healing rates in both groups after 8 weeks, with mean healing times of 5 weeks for collagen and 6 weeks for hydrocolloids. Both groups also had comparable daily healed areas (6 mm^2^/day). The study concluded that collagen and hydrocolloid therapies are equally effective for treating pressure ulcers.

Another randomized, controlled pilot study conducted at RWTH University Hospital in Aachen compared collagen and foam dressings for stagnating pressure ulcers (Piatkowski et al., 2012). Ten patients were enrolled, with five receiving a foam dressing (Suprasorb P; Lohmann and Rauscher) (Group A) and five receiving a collagen dressing (Suprasorb C; Lohmann and Rauscher) covered by the same foam dressing (Group B). The study found that wound fluid from Group B (collagen + foam) significantly improved angiogenesis (p < 0.05) compared to Group A (foam only). Group B also showed faster and greater increases in TIMP-1 and TIMP-2 levels, along with a quicker and more pronounced decline in MMP-2, MMP-9 (p < 0.04), and elastase levels. These findings indicate a more rapid reduction in inflammation. While both groups experienced healing, Group B demonstrated faster progress, highlighting the therapeutic benefits of collagen dressings for pressure ulcers.

### 4.3 Chitosan

Chitosan is highly biodegradable, leaving no residue in the body and minimizing the risk of secondary damage or patient discomfort (Wang et al., 2020). Its natural origin ensures excellent biocompatibility with human tissues, making it suitable for a wide range of patients (Tang et al., 2023). Chitosan wound dressings also have strong antimicrobial properties. They inhibit the growth of pathogens, reduce the risk of infection, and accelerate the healing process (Matica et al., 2019; Moeini et al., 2020). This antimicrobial effect is especially valuable for pressure ulcer patients, as it helps prevent secondary infections and improves overall outcomes. In addition, chitosan dressings maintain a moist environment that supports tissue regeneration (Liu et al., 2018). By stimulating cell growth and repair around the wound, they speed up healing, shorten recovery time, improve outcomes, and reduce complications.

**FIGURE 4:**
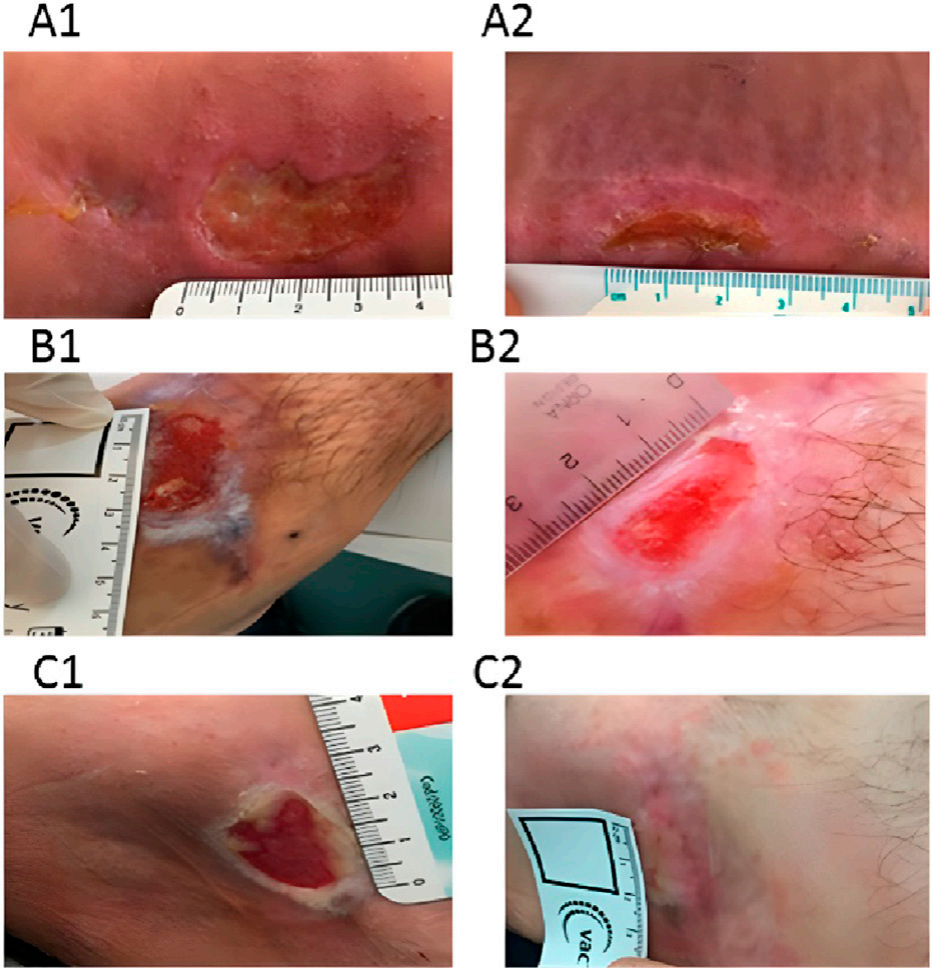
The images illustrate the condition of pressure ulcers prior to treatment **(A1, B1, C1)** and following a 30-day course of chitosan gel therapy **(A2, B2, C2)**. Reproduced with permission from Campani et al. (2018). Copyright ^©^ 2018 The Authors.

The clinical benefits of chitosan dressings for pressure ulcers are well-documented (Campani et al., 2018). In a pilot study, Campani et al. used chitosan gel to treat pressure ulcers and reported significant reductions in lesion size. Healing was observed in 90% of participants (Campani et al., 2018) (Figure 4). The study also emphasized the cost-effectiveness of the gel, which was prepared at minimal expense in the hospital pharmacy’s sterile area, significantly lowering patient care costs.

A multicenter, prospective, randomized, open-label clinical study at three Chinese medical centers compared the safety and efficacy of Next-Generation KA01 chitosan wound dressings with standard gauze (T.V. Gauze) for non-healing chronic wounds, including pressure ulcers, vascular ulcers, diabetic foot ulcers, and minimally infected or at-risk wounds (Mo et al., 2015). Ninety patients participated (45 per group). After 4 weeks, the chitosan group showed significantly greater wound size reduction (65.97% ± 4.48%) compared to the control group (39.95% ± 4.48%). Pain levels were also significantly lower in the chitosan group (1.12 ± 0.23) versus the control group (2.30 ± 0.23). These results suggest that next-generation chitosan dressings enhance wound healing by promoting re-epithelialization and reducing pain.

Another study assessed the efficacy of chitosan-based hydrocolloid dressings in 80 patients with chronic refractory wounds, including pressure ulcers (Liu and Shen, 2022). Patients were randomized into two groups: one treated with chitosan-based hydrocolloid dressings (40 patients) and the other with inert saline gauze (40 patients). After 3 weeks, the chitosan group showed significantly better wound healing, reduced pain levels, and fewer symptoms of itching compared to the control group (P < 0.05). Wound area reductions and overall healing efficiency were also significantly higher in the chitosan group. Additionally, dressing changes were less frequent, and total treatment costs were lower in the chitosan group (P < 0.05), further supporting its potential as an effective and economical option for pressure ulcer care. These findings are consistent with a separate randomized controlled trial conducted in Iran, which also confirmed the efficacy of chitosan dressings in treating pressure ulcers (Kordestani et al., 2008).

### 4.4 Poly (lactic-co-glycolic acid)

Poly (lactic-co-glycolic acid) (PLGA) is a biodegradable polymer that serves as a safe and effective dressing for pressure ulcers, thanks to its excellent biocompatibility, controlled release properties, and biodegradability (Chereddy et al., 2016). The composition and molecular weight of PLGA can be tailored to regulate drug release, ensuring consistent and effective therapy for pressure ulcer patients and improving treatment outcomes (Jiang et al., 2021; Choipang et al., 2018; Liu et al., 2020). By incorporating antimicrobial agents and growth factors, PLGA dressings can effectively prevent infections and promote wound healing, offering innovative solutions for pressure ulcer management (Choipang et al., 2018; Liu et al., 2020; Catanzano et al., 2021; Park et al., 2017). Its biodegradability allows PLGA to break down gradually into lactic acid and glycine, which are naturally metabolized by the body. This prevents secondary injuries, reduces adverse effects, and enhances patient comfort and safety. Moreover, PLGA dressings provide excellent mechanical properties and adaptability. They can be customized to fit various wound sizes and shapes, offering superior protection, reducing infection risk, and promoting faster healing.

### 4.5 Polyurethane

Polyurethane wound dressings have shown significant advantages and progress in pressure ulcer care. As a novel material, polyurethane offers excellent biocompatibility, high absorbency, superior elasticity, and adjustable physicochemical properties (Cui et al., 2023), making it an effective solution for treating pressure ulcers. The biocompatibility of polyurethane ensures its safety for a wide range of patients, including those with allergies to other dressing materials, addressing the need for personalized care. Its exceptional absorbency manages wound exudate efficiently, maintaining optimal moisture levels for cell growth and healing (Samatya Yılmaz and Aytac, 2023). By retaining moisture without peeling or drying out, polyurethane dressings protect wounds and reduce discomfort caused by frequent dressing changes, a critical factor in managing pressure ulcers. Polyurethane’s elasticity allows the dressings to conform to changing wound shapes, minimizing mechanical irritation and reducing the risk of further ulcer development (Wang et al., 2024; Abdullah et al., 2023; Pongmuksuwan and Harnnarongchai, 2022). This feature benefits both active and bedridden patients by improving comfort and reducing the likelihood of complications. Additionally, the adaptable physicochemical properties of polyurethane can be tailored to specific needs. Modifying its composition and structure enables adjustments in breathability, hydrophilicity, and degradability, addressing various pressure ulcer scenarios (Sikdar et al., 2022). These features position polyurethane as a promising material for future advancements in pressure ulcer treatment.

Clinical trials strongly support the efficacy of polyurethane wound dressings for managing and preventing pressure ulcers. Dutra et al. investigated the performance of transparent polyurethane film (PF) compared to hydrocolloid dressings (HD) in preventing pressure ulcers (Dutra et al., 2015). In a study with 160 patients, the PF group required significantly fewer dressing changes, especially in the sacral region, suggesting superior performance of polyurethane films. Further research highlighted the lower per-change cost of polyurethane films compared to hydrocolloid dressings (Dutra et al., 2016), enhancing their cost-effectiveness. Additionally, transparent polyurethane films have been shown to effectively prevent heel pressure ulcers (Souza et al., 2013). Polyurethane foam dressings are also widely used for treating pressure ulcers. A randomized controlled trial compared a novel polyurethane foam to standard foam in negative pressure wound therapy. The novel foam was equally effective but easier to remove, with less fragmentation and residue left in wounds (Wagstaff et al., 2014). It also prevented inward margin growth, reducing trauma during dressing changes and minimizing bleeding from granulation tissue. These findings confirm the safety and biocompatibility of the novel polyurethane foam, supported by earlier animal studies (Greenwood et al., 2010; Greenwood and Dearman, 2012). Furthermore, trials have demonstrated the efficacy of polyurethane foam dressings in preventing sacral pressure ulcers in elderly hip fracture patients (Forni et al., 2018; Forni et al., 2022).

In conclusion, polyurethane wound dressings offer significant advantages in pressure ulcer care. Their biocompatibility, absorbency, elasticity, and adjustable physicochemical properties provide effective and comfortable solutions for patients. Clinical validation demonstrates the progress made in pressure ulcer treatment using polyurethane dressings, contributing to improved patient quality of life. Continued advancements in medical technology promise an even greater role for polyurethane wound dressings in pressure ulcer care.

## 5 Challenges and prospects

Advanced biomaterials hold great promise for preventing and treating pressure ulcers, but several challenges must still be addressed. First, biocompatibility is crucial. Patient responses can vary, sometimes leading to immune or toxic reactions. To ensure safety, thorough biocompatibility testing—both *in vitro* and in vivo—is essential. Using natural biomaterials, modifying surfaces, and adopting other biocompatible strategies can help reduce these risks. Another challenge is optimizing mechanical properties. Biomaterials need to be strong yet flexible enough to provide proper support and protect wounds. Achieving this balance requires fine-tuning material composition, structure, and fiber alignment, or using composite materials designed for these purposes. Controlling degradation rates is also critical. If a material breaks down too quickly, the wound may lose protection. If it degrades too slowly, healing may be delayed. Adjustments to material composition, microstructure, and degradation mechanisms can help ensure materials degrade at the right pace and integrate seamlessly with surrounding tissues. Cost-effective production is another major factor. To make these materials more accessible, manufacturing must be efficient and affordable. Advances in automation, mass production, and novel synthesis methods will be key to lowering costs without compromising quality. Finally, clinical translation and regulatory approval remain significant hurdles. Moving from lab research to real-world applications requires rigorous clinical trials and navigating complex regulatory processes. Success in this area depends on careful trial design, interdisciplinary collaboration, and close cooperation with regulatory agencies. By overcoming these challenges, advanced biomaterials can see wider use in pressure ulcer care. This will lead to more effective treatments and better outcomes for patients. With ongoing innovation and experience, these materials will continue to evolve, playing an even greater role in improving medical care.

Future research in this field will focus on multifunctional composites, smart materials, and personalized treatments to address current challenges and improve effectiveness. Multifunctional composites are designed to combine multiple properties into a single material. These properties include antimicrobial activity, healing promotion, and monitoring capabilities. Such materials can prevent infections, speed up healing, and track progress in real time, offering a comprehensive solution for pressure ulcer care. Smart materials will focus on responsiveness and controlled drug delivery. These materials can change their properties in response to environmental factors or specific stimuli. Controlled release systems will allow precise delivery of drugs or therapeutic substances, paving the way for more targeted and effective treatments. Personalized treatments will adapt care to the unique needs of each patient. By tailoring biomaterial selection and treatment plans to individual characteristics, these strategies will ensure more precise and effective outcomes. By combining these approaches, future research aims to create comprehensive and personalized solutions for pressure ulcer prevention and treatment. Continued innovation and collaboration across disciplines will further enhance the role of advanced biomaterials, improving patient outcomes and quality of life.

## 6 Conclusion

Advancements in biomaterials for pressure ulcer prevention and treatment bring new hope and opportunities for patients. Ongoing research and innovation hold the potential to transform pressure ulcer care, enabling more effective treatment strategies, better patient outcomes, and an enhanced quality of life. To achieve these breakthroughs, prioritizing the development of novel biomaterials and fostering interdisciplinary collaboration will be essential. This focus will accelerate the clinical translation of these technologies, ultimately improving medical care and treatment experiences for individuals affected by pressure ulcers.
